# Electrocardiographic changes in spondyloarthritis and use of anti-TNF-α drugs: a retrospective study with 100 patients

**DOI:** 10.31744/einstein_journal/2019AO4539

**Published:** 2019-03-18

**Authors:** Betânia Longo, Luziel Andrei Kirchner, Juliana Simioni, Ana Paula Beckhauser de Campos, Thelma Larocca Skare

**Affiliations:** 1 Serviço de Reumatologia, Hospital Universitário Evangélico de Curitiba, PR, Brazil.; 2 Serviço de Cardiologia, Hospital Cardiológico Costantini, Curitiba, PR, Brazil.

**Keywords:** Spondylarthropathies, Electrocardiography, Bundle-branch block, HLA B27 antigen, Anti TNF-α, Tumor necrosis factor-alpha, Espondiloartropatias, Eletrocardiografia, Bloqueio de ramo, Antígeno HLA-B27, Anti-TNF-α, Fator de necrose tumoral alfa

## Abstract

**Objective:**

To investigate the prevalence of electrocardiographic changes in patients with spondyloarthritis and to correlate these changes with use of anti-tumor necrosis factor-alpha (TNF-α) drugs and HLA-B27 positivity.

**Methods:**

Retrospective study including 100 patients diagnosed with spondyloarthritis according to Assessment of SpondyloArthritis International Society (ASAS) criteria and 50 controls. Epidemiological and clinical features, results of inflammatory activity tests, HLA-B27 positivity, and medication use data were extracted from medical records. Disease activity was assessed using the Bath Ankylosing Spondylitis Disease Activity Index (BASDAI). All participants were submitted to electrocardiogram performed using a 12-lead device; rhythm, heart rate, conduction disorders and QT interval corrected using the Bazett formula were analyzed.

**Results:**

Of 100 patients with spondyloarthritis, 51 were on anti-TNF-α drugs and 49 were not. HLA-B27 was detected in 53.1% of patients in the sample. Patients with spondyloarthritis had lower heart rate (p=0.06), longer QT interval (p<0.0001) and higher prevalence of right bundle branch block (p=0.014) compared to controls. Duration of disease was weakly correlated with heart rate (Rho=0.26; 95%CI: 0.06-0.44; p=0.008). The prevalence of right bundle branch block was positively correlated with HLA-B27 positivity. Use of Anti-TNF-α drugs did not interfere with electrocardiographic parameters.

**Conclusion:**

Patients with spondyloarthritis had lower heart rate, longer QT interval and a higher prevalence of right bundle branch block compared to controls. HLA-B27 positivity was associated with the prevalence of right bundle branch block. Anti-TNF-α drugs had no impact on electrocardiographic findings.

## RESUMO

**Objetivo:**

Avaliar a prevalência de alterações eletrocardiográficas em pacientes com espondiloartrites, correlacionando-as com o uso de medicações antifator de necrose tumoral alfa (TNF-α) e presença do HLA-B27.

**Métodos:**

Estudo retrospectivo com 100 pacientes com diagnóstico de espondiloartrites pelo critério *Assessment of SpondyloArthritis International Society* (ASAS) e 50 controles. Foram coletados nos prontuários dos pacientes, dados epidemiológicos, clínicos, exames de atividade inflamatória, presença do HLA-B27, e uso de medicamentos. A atividade de doença foi avaliada pelo *Bath Ankylosing Spondylitis Disease Activity Index* (BASDAI). Todos foram submetidos a eletrocardiograma realizado com aparelho de 12 derivações, sendo analisados ritmo, frequência cardíaca, distúrbios de condução e intervalo QT corrigido pela fórmula de Bazett.

**Resultados:**

Dos 100 pacientes com espondiloartrites, 49 não usavam anti-TNF-α e 51 utilizavam este medicamento. O HLA-B27 estava presente em 53,1% da amostra. A frequência cardíaca foi mais baixa (p=0,06), o intervalo QT foi mais prolongado (p<0,0001) e existia mais perturbação de condução do ramo direito (p=0,014) nos pacientes com espondiloartrites do que nos controles. Uma modesta correlação de tempo de doença com frequência cardíaca foi encontrada (Rho=0,26; IC95%: 0,06-0,44; p=0,008). A presença do HLA-B27 aumentou a prevalência de perturbação de condução do ramo direito. Nenhum dos parâmetros eletrocardiográficos analisados alterou-se com uso de anti-TNF-α.

**Conclusão:**

Pacientes com espondiloartrites tiveram frequência cardíaca menor, maior intervalo QT e prevalência maior de perturbação de condução do ramo direito do que controles. O HLA-B27 influi no aparecimento de perturbação de condução do ramo direito. O uso de anti-TNF-α não influiu nos achados eletrocardiográficos.

## INTRODUCTION

Spondyloarthritis (SpA) comprise a group of chronic diseases including ankylosing spondylitis (AS), psoriatic arthritis (PsA), reactive arthritis (ReA), juvenile SpA, inflammatory bowel disease-related SpA and undifferentiated spondyloarthritis.^(^ ^1^ ^,^ ^2^ ^)^ Major complaints associated with SpA are of musculoskeletal origin; however, several extra-articular manifestations have been reported, including cardiac conditions such as aortitis, aortic insufficiency, conduction disorders and atrioventricular blocks,^(^ ^1^ ^)^ which are thought to affect approximately 10% of patients. Cardiac involvement is often subclinical, associated with longstanding disease^(^ ^1^ ^,^ ^3^ ^)^ and unrelated to articular disease activity.^(^ ^1^ ^)^ Pacemaker implantation and valve replacement may be required in severe cases.^(^ ^4^ ^)^ Ankylosing spondylitis is the best understood SpA in terms of cardiac manifestations, which also include atrioventricular blocks (AVBs), arrhythmias and ventricular dysfunction due to fibrosis. First-degree AVBs are associated with an increased risk of atrial fibrillation (AF),^(^ ^5^ ^)^ which in turn is associated with stroke, heart failure and increased mortality.^(^ ^6^ ^)^

Supraventricular arrhythmias were the most common findings in an European study^(^ ^7^ ^)^ with AS patients. In that study, prolonged P wave duration and dispersion were associated with inflammatory activity, as measured using the Bath Ankylosing Spondylitis Disease Activity Index (BASDAI).

The B27 human leucocyte antigen (HLA) is strongly associated with SpA (particularly AS) and is found in up to 90% of cases.^(^ ^3^ ^)^ Third-degree AVBs of unknown cause requiring pacemaker implantation in young males are thought to be associated with HLA-B27,^(^ ^1^ ^)^ and 15 to 20% of individuals with permanent pacemaker are positive for this antigen.^(^ ^3^ ^)^ However, failure to detect associations between the HLA-B27 positivity and conduction abnormalities Forsblad-d’Elia et al.^(^ ^4^ ^)^ raised controversies over this matter.

Tumor necrosis factor alpha (TNF-α) is a pro-inflammatory cytokine playing a significant role in SpA pathogenesis. Pharmacological agents known as anti-TNFα are able to block this inflammatory mechanism and may be used for SpA treatment.^(^ ^8^ ^)^

In spite of several studies investigating the impact of anti-TNF-α drugs on heart failure and atherogenesis associated with chronic rheumatoid diseases, little is known about the impact of these drugs on conduction disorders.

## OBJECTIVE

To investigate the prevalence of electrocardiographic changes in patients with spondyloarthritis and correlations between electrocardiographic changes, use of anti-TNF-α drugs and HLA-B27 positivity.

## METHODS

Retrospective study based on a convenience sample comprising patients seen at the outpatient rheumatology service of *Hospital Universitário Evangélico de Curitiba* between 2016 and 2017. Patients aged over 18 years and diagnosed with SpA according to Assessment of SpondyloArthritis International Society (ASAS)^(^ ^9^ ^)^ criteria were included. This study was approved by the Ethics and Research Committee of *Sociedade Evangélica Beneficente de Curitiba* , opinion No. 1.779.523, CAAE: 60727616.9.0000.0103, and was exempt from informed consent.

Medical records were screened for epidemiological and clinical data, presence of comorbidities, erythrocyte sedimentation rate (ESR) values, C-reactive protein (CRP) levels and HLA-B27 positivity. Disease activity was assessed using the Bath Ankylosing Spondylitis Disease Activity Index (BASDAI).^(^ ^10^ ^)^ This instrument rates fatigue, spinal pain, peripheral arthritis, enthesytis and morning stiffness severity and duration^(^ ^7^ ^)^ on a zero to 10 scale; scores ≥4 are suggestive of active disease.^(^ ^10^ ^)^ Medications prescribed were recorded and patients allocated to one of two groups according to use of anti-TNF-α drugs ( *i.e.* , treated or not treated) and compared.

The Control Group comprised patients asymptomatic for rheumatoid diseases, who sought Internal Medicine Department for check-up purposes, whose medical records included the same tests. Groups were matched according to sex, age, smoking habits and history of hypertension and *diabetes mellitus* .

Exclusion criteria were as follows: pregnant women, patients suffering from chronic renal failure, chronic liver disease, neurologic diseases, structural cardiac disease in patients taking drugs acting on the autonomic nervous system, and patients with missing data.

Electrocardiograms (ECG) were systematically performed by a single examiner following a 15-minutes rest period and using 12-lead ECG system at 25mm/sec velocity and 10mm/mV gain setting. QT intervals were corrected (QTc) for heart rate using the Bazett formula.^(^ ^11^ ^,^ ^12^ ^)^ Incomplete rigth bundle branch blocks (IRBBB) assessment was based on the following parameters: QRS enlargement ≥0.12s, slurred S waves in leads D1, aVL, V5 and V6, qR waves in lead aVR with R wave slurring, rSR’ or rsR’ in lead V1 with thick R’, variable QRS electrical axis tending to the right in the frontal plane and asymmetrical T wave opposite to terminal QRS delay.^(^ ^13^ ^)^ Electrocardiograms were read by a single investigator blinded to clinical data.

QTc interval normality thresholds were set at 0.46 seconds (460 milliseconds) and 0.47 seconds (460 milliseconds) for male and female patients, respectively. Normal heart rate was defined as 50 to 100bpm.

Data were compiled in frequency and contingency tables. Measures of central tendency were expressed as mean and standard deviation, or median and interquartile ranges (Gaussian and non-Gaussian distribution, respectively). Sample normality was investigated using the Shapiro-Wilk test. Nominal and numerical data were compared using the Fisher or the χ ^2^ and the Student’s *t* or the Mann-Whitney test, respectively. Correlations were investigated using the Spearman test. The level of significance was set at 5% (p=0.05). Calculations were made sing Medcalc10.0 software.

## RESULTS

The sample comprised 150 individuals, as follows: 51 diagnosed with SpA and using anti-TNF-α drugs, 49 diagnosed with SpA and not using anti-TNF-α drugs and 50 controls. Patient data are shown in table 1 .

**Table 1:** Descriptive data of 100 patients diagnosed with spondyloarthritis included in the sample

Descriptive data	n=100
SpA manifestation	
Ankylosing spondylitis	53
Psoriatic arthritis	31
Undifferentiated	7
IBD-related	4
Juvenile SpA	2
Reactive arthritis	2
Non radiographic SpA	1
Involvement	
Axial	30
Peripheral	19
Axial and peripheral	51
Sex	
Male	53
Female	47
Age, years	16-73; mean 48.9±11.9
Duration of disease, years	1-30; median 7; IQR=3.2-12.0
HLA-B27 positive	42*
Medications	
Non-steroidal anti-inflammatory drugs	43
Methotrexate	25
Leflunomide	51
Anti-TNF-α	51
Etanercept	17
Adalimumab	20
Infliximab	14
BASDAI	0-8, median 2.0 (IQR=1.0-3.4)
C-reactive protein, mg/dL	0-43, median 6.0 (IQR=2.3-6.0)
ESR, mm	1-100, median 16.5 (IQR=6.2-26.7)

* Data available in 79 medical records only. IBD: inflammatory bowel disease; BASDAI: Bath Ankylosing Spondylitis Disease Activity Index; IQR: interquartile range; ESR: erythrocyte sedimentation rate; SpA: spondyloarthritis.

Comparative analysis revealed similar findings between SpA and control patients regarding sex (male/female ratio, 57% *versus* 58%; p=0.90), age (mean age 48.9±11.9 *versus* 46.0±13.4 years; p=0.19), smoking habits (21% *versus* 28%; p=0.33), alcohol abuse (10% per group; p=1.0) and prevalence of hypertension (32% *versus* 36%; p=0.46), *diabetes mellitus* (10% *versus* 4%; p=0.21) and hypothyroidism (14% *versus* 8%; p=0.42).

Comparative analysis of electrocardiographic findings in patients and controls are shown in table 2 . Patients suffering from SpA had lower heart rate and higher prevalence of IRBBB compared to controls. Spondyloarthritis patient had more prolonged QT intervals; QTc tended to be more prolonged in patients with SpA compared to controls.

**Table 2:** Comparative analysis of electrocardiographic findings in patients with spondyloarthritis and controls

ECG	SpA Group (n=100)	Control Group (n=50)	p value
Sinus rhythm, n (%)	100 (100)	50 (100)	
Heart rate, bpm	47-96, median 68 (59-76)	51-101, median 73.5 (66.7-82.0)	0.006
Heart rate, seconds	0.61-1.28, mean 0.90±0.14	0.59-1.18, mean 0.83±0.12	0.009
First-degree atrioventricular block, n (%)	3 (3)	0	0.55
Ventricular repolarization changes, n (%)	54 (54)	20 (40)	0.10
Right bundle branch block, n (%)	37 (37)	6 (12)	0.014*
QT, seconds	0.32-0.49, median 0.40 (0.37-0.40)	0.32-0.48, median 0.36 (0.36-0.40)	<0.0001
QTc, seconds	0.36-0.49, median 0.41 (0.39-0.43)	0.34-0.49, median 0.40 (0.38-0.43)	0.08

* Odds ratio: 4.3; 95% confidence interval 1.67-11. ECG: electrocardiogram; QTc: corrected QT interval (Bazett formula).

Duration of cardiac disease and heart rate were weakly correlated (p=0.008, Rho=0.26; 95% confidence interval − 95%CI: 0.06–0.44). The prevalence of IRBBB was positively correlated with HLA-B27 positivity (p=0.03); HLA-B27 positivity was not associated with disease activity as measured using BASDAI, CRP and ESR (non-significant p values).

Relations between remaining electrocardiographic changes and HLA-B27 positivity are shown in table 3 .

**Table 3:** Electrocardiographic characteristics and HLA-B27 positivity in the group of patients with spondyloarthritis

Characteristic	HLA-B27 positive (n=42)	HLA-B27 negative (n=37)	p value
Heart rate, bpm	47-94, mean 67.5±12.4	50-97, mean 69.2±10.0	0.52
Heart rate, duration in seconds	0.64-1.28, mean 0.91±0.16	0.62-1.2, mean 0.88±0.12	0.33
QT, seconds	0.32-0.49, mean 0.39±0.03	0.32-0.44, mean 0.38±0.02	0.70
QTc, seconds	0.38-0.49, median 0.41 (0.39-0.42)	0.36-0.48, median 0.42 (0.40-0.43)	0.12

QTc: corrected QT interval (Bazett formula).

According to comparative analysis of electrocardiographic data, anti-TNF-α drugs had no impact on electrocardiographic findings ( Table 4 ).

**Table 4:** Comparative analysis of electrocardiographic data from patients with spondyloarthritis treated or not treated with anti-TNF-α drugs

Characteristic	Treated with anti-TNF-α n=51	Not treated with anti-TNF-α n=49	p value
Heart rate, bpm	47-98, median 68 (58-77)	47-97, median 68 (60-75)	0.77
Heart rate, duration in seconds	0.61-1.28, mean 0.90±0.15	0.62-1.28, mean 0.89±0.14	0.71
QT, seconds	0.32-0.49, median 0.40 (0.37-0.40)	0.32-0.46, median 0.40 (0.37-0.40)	0.62
QTc, seconds	0.36-0.48, mean 0.41±0.02	0.36-0.49, mean 0.41±0.025	0.53
Incomplete right bundle branch block, n/n total	20/51	17/49	0.63

QTc: corrected QT interval (Bazett formula).

Three male patients with SpA had QTc intervals ≥0.46 seconds, two of whom were on anti-TNF-α drugs. No QTc changes were detected in control patients.

## DISCUSSION

This study revealed lower heart rates, increased prevalence of IRBBB, and higher QTc values in patients suffering from SpA compared to controls. Normal or above normal QTc intervals were detected in three patients. With the exception of IRBBB prevalence, these changes were not associated with HLA-B27 positivity. Use of anti-TNF-α drugs had no impact on the frequency of these findings.

The sample in this study has some particularities. Firstly, patients suffering from different forms of SpA were included, while most other studies are limited to patients with AS.^(^ ^3^ ^,^ ^4^ ^,^ ^7^ ^,^ ^10^ ^)^ Spondyloarthritis is associated with higher prevalence of HLA-B27 positivity;^(^ ^3^ ^)^ therefore lower prevalence of this histocompatibility antigen in this sample may explain the lack of associations with electrocardiographic changes reported by other authors. A second explanation, also related to lower prevalence of HLA-B27 positivity, is the fact that this study was based on a Brazilian population. The prevalence of HLA-B27 positivity is lower in the Brazilian compared to Caucasian populations due to the high levels of local racial miscegenation.^(^ ^1^ ^)^

Anti-TNF-α drugs did not interfere with electrocardiographic findings, including IRBBB. Two anedoctal theories may explain conduction disorders in the context of this study: atrioventricular node abnormalities and intraventricular septal inflammation.^(^ ^3^ ^)^ Endarteritis of small vessels supplying the aortic valve and atrioventricular node and resulting obliteration by inflammatory process (very similar to that observed in joints) has been described.^(^ ^4^ ^)^ Had electrocardiographic changes resulted from the inflammatory process, it would be reasonable to assume such changes would be reverted by anti-TNF-α drugs. Findings of this study do not support this hypothesis. However, given the relatively long duration of disease in this sample, inflammatory changes may have progressed to irreversible, fibrotic changes. Weak as they may be, correlations between duration of disease and IRBBB have been detected in this study. Investigations with recently diagnosed patients treated with anti-TNF-α drugs may help to clarify this issue.

Lower heart rates were recorded in SpA patients in this study. Similar findings have been reported by Dik et al.,^(^ ^14^ ^)^ who detected bradycardia in 30% of 130 patients suffering from AS. Autonomic dysfunctions, particularly parasympathetic, have been described in patients with psoriatic arthritis^(^ ^15^ ^)^ and may explain this finding. Bradycardia may also reflect other conduction disorders.^(^ ^12^ ^)^

This study also revealed a tendency towards longer QTc intervals regardless of anti-TNF-α drug use. In contrast, DI Franco et al.,^(^ ^16^ ^)^ reported asymptomatically increased QT interval and dispersion in patients suffering from inflammatory polyarthritis treated with anti-TNF-α drugs. Among patients with SpA in this sample, three had above normal QTc intervals, which has been associated with increased risks of *torsade de pointes* type tachycardia *.* ^(^ ^12^ ^)^ Of these, only one was on anti-TNF-α drugs.

This study has several limitations, such as lack of HLA-B27 data in control individuals and small sample size. Retrospective design introduced yet another limitation, as duration of anti-TNF-α treatment could not be accounted for. Prospective studies are warranted to further investigate the matter. Still, findings of this study emphasize the high prevalence of electrocardiographic changes in patients suffering from SpA, who are already prone to cardiovascular problems given the chronic, inflammatory nature of the disease,^(^ ^17^ ^)^ and therefore deserve closer medical attention.

## CONCLUSION

Patients suffering from spondyloarthritis had lower heart rate, longer corrected QT interval and higher prevalence of incomplete right bundle branch block compared to controls. Anti-TNF-α medication had no impact on electrocardiographic findings.
